# New Poly(β-Cyclodextrin)/Poly(Vinyl Alcohol) Electrospun Sub-Micrometric Fibers and Their Potential Application for Wastewater Treatments

**DOI:** 10.3390/nano10030482

**Published:** 2020-03-07

**Authors:** Anastasia Anceschi, Fabrizio Caldera, Moira Bertasa, Claudio Cecone, Francesco Trotta, Pierangiola Bracco, Marco Zanetti, Mery Malandrino, Peter E. Mallon, Dominique Scalarone

**Affiliations:** 1 Department of Chemistry and NIS Centre, University of Turin, Via P. Giuria 7, 10125 Torino, Italy; anastasiaandrea.anceschi@unito.it (A.A.); fabrizio.caldera@unito.it (F.C.); moira.bertasa@unito.it (M.B.); claudio.cecone@unito.it (C.C.); francesco.trotta@unito.it (F.T.); pierangiola.bracco@unito.it (P.B.); marco.zanetti@unito.it (M.Z.); mery.malandrino@unito.it (M.M.); 2 ICxT Centre, University of Turin, Lungo Dora Siena 100, 10153 Torino, Italy; 3 Department of Chemistry and Polymer Science, Stellenbosch University, De Beers Street, Stellenbosch 7600, South Africa; pemallon@sun.ac.za

**Keywords:** polycyclodextrin, polysaccharides, electrospinning, fibers, heavy metals, sorbent materials, wastewater

## Abstract

Cyclodextrin (CD)-based polymers are known to efficiently form molecular inclusion complexes with various organic and inorganic guest compounds. In addition, they also have a great potential as metal complexes because deprotonated hydroxyls can strongly bind metal ions under alkaline conditions. The range of environmental conditions for polycyclodextrin/metal ion complexation can be extended by the polymerization of CDs with polyacids. This article describes the preparation and characterization of a new type of poly(β-cyclodextrin) (Poly-βCD) sub-micrometric fibers and explores their potential as metal ion sorbents. A water-soluble hyper-branched β-cyclodextrin polymer was blended with poly(vinyl alcohol) (PVA) and here used to improve the mechanical and morphological features of the fibers. Solutions with a different Poly-βCD/PVA ratio were electrospun, and the fibers were cross-linked by a post-spinning thermal treatment at 160 °C to ensure non-solubility in water. The fiber morphology was analyzed by scanning electron microscopy (SEM) before and after the curing process, and physical-chemical properties were studied by Fourier transform infrared spectroscopy (FTIR), thermogravimetric analysis (TGA) and differential scanning calorimetry (DSC). The capability of the insoluble cyclodextrin-based fibers to remove heavy metals from wastewaters was investigated by testing the adsorption of Cu^2+^ and Cd^2+^ using inductively coupled plasma-optical emission spectroscopy (ICP-OES). The results suggest that the poly(β-cyclodextrin)/poly(vinyl alcohol) sub-micrometric fibers can complex metal ions and are especially effective Cu^2+^ sorbents, thus opening new perspectives to the development of fibers and membranes capable of removing both metal ions and organic pollutants.

## 1. Introduction

Industrial wastewaters are often contaminated by a variety of compounds that can have negative effects on the environment [1]. A large amount of heavy metals, such as Cd, Cr, Zn, Ni, Pb, and Cu, is discharged by industries in wastewater [2,3]. One of the problems related with the presence of heavy metals in water is that they are not biodegradable in an aquatic environment and so they can be absorbed by aquatic organisms and, therefore, enter the food chain. Copper is naturally present in the human body and it plays an important role in enzyme synthesis or for the development of tissues and bones [4]. However, an excess of Cu^2+^ is toxic and cancerogenic. Its deposition in the human body causes a succession of serious effects such as headache, liver and kidney failure, and gastrointestinal bleeding. Indeed, the World Health Organization (WHO) sets the limits of Cu^2+^ in drinking water at 1.5 mg/L [5]. Another teratogenic and cancerogenic heavy metal is cadmium. It is found in industrial discharge, such as the manufacturing of batteries, fertilizers, pigments, and electroplating [6]. Furthermore, it is naturally present as a minor constituent of some ores and coal deposits [7]. The limit imposed by WHO for cadmium in drinking water is 3.0 µg/L [8].

Many technologies are being evaluated for copper and cadmium removal from wastewaters. One of the commonly applied methods is by chemical precipitation [9], which involves the use of hydroxides, carbonates, or sulfates to form insoluble precipitates [10]. This process is the best for treating wastewater with a high concentration of metals, but it is almost ineffective when the concentration of heavy metals is low [11]. Other methods for industrial wastewater treatments employ chelating agents [12] or ion exchange resins [13]. Adsorption is also a well-established technique to remove heavy metals from water. It offers many advantages, such as flexibility in design, reversibility, and ease in regeneration and therefore in reuse [11]. Many adsorption materials have been found to effectively adsorb Cu^2+^ and Cd^2+^ [14], including non-conventional low-cost adsorbents such as polysaccharide-based materials [15]. Adsorbent materials are also used to produce membranes. In fact, membrane science is constantly expanding, and recently, new materials and technologies for the fabrication of membranes have been proposed [16]. Membranes can be considered as barriers that are able to separate two distinct phases. Therefore, a good membrane is able to perform a separation, blocking one or more pollutants in a phase (feed) and transporting the remaining species toward the other phase (permeate). The most commonly used techniques to synthesize membranes are by phase inversion, interfacial polymerization, track-etching, casting, and electrospinning [17,18,19].

In the last decade, electrospinning has become very popular. It is a technique by which high voltages are used to produce an interconnected network of small fibers with diameters ranging from nanometers to the microscale. Moreover, electrospinning allows for the production of fibers from a variety of polymer/solvent systems [20]. Over the years, more than 200 different types of polymers have been successfully electrospun [21,22]. In particular, the advantages and limits of the electrospun fibrous membranes proposed so far for the removal of heavy metals have been discussed in a few publications, reviewing the natural and synthetic polymers that are capable of forming complexes with metal ions, as well as strategies for improving the complexation efficiency, such as blending, the surface modification of functional groups, and the incorporation of functional nanoparticles [23,24].

Recently, particular attention has been focused on membranes from polysaccharides since polysaccharides are naturally abundant, inexpensive, easily available, and are a renewable resources [25]. The versatility of polysaccharides is attributed to their intrinsic properties such as the presence of reactive functional groups and the flexible structure of the chain [26]. In particular, starch is one of the most used polysaccharides. It has a large number of reactive groups allowing chemical modifications that make it suitable for many applications. Important derivatives of starch are cyclodextrins (CDs) [15]. Cyclodextrin-based polymers have been proposed for the abatement of pollutants from water. CDs possess a toroid structure with a lipophilic inner surface and a hydrophilic outer surface that make them efficient materials for the formation of inclusion complexes with many organic and inorganic molecules. Moreover, CDs easily react with cross-linking agents to give hyper-cross-linked polymers that are water-insoluble. The use of these kinds of cross-linked polymers for the abatement of pollutants in wastewaters is known [27,28]. The most common cross-linking agent is epichlorohydrin, although other bi- and polyfunctional chemicals have been proposed such as diisocyanates and polyacids or anhydrides [29,30]. Moreover, β-CD polymers cross-linked with polyacids or anhydrides present carboxylate moieties that can be exploited for heavy metal complexation [31]. In particular, the study reported by Euvrard et al. demonstrates that CD-based polymers cross-linked by polycarboxylic acids are materials that can potentially remove at the same time both metal ions and organic pollutants, such as polycyclic aromatic hydrocarbons and alkylphenols [32].

Trotta et al. reported the synthesis of a hyper-branched water-soluble β-CD polymer (Poly-βCD) obtained by reacting β-CD with pyromellitic dianhydride (PMDA) beyond the critical conditions that allow the phenomenon of gelation to occur [33]. The capability of the hyper-branched polymer to form inclusion complexes with organic molecules was tested by using fluorescein as a probe molecule. Importantly, being water-soluble, this hyper-branched polymer can be processed via the electrospinning of water solutions to produce micrometric fibers. Then, these fibers can be easily made insoluble through a post-spinning cross-linking thermal treatment [34] and used for the controlled release of organic molecules [35]. Furthermore, the presence of free carboxylic groups that are potentially capable of complexing metal ions makes these fibers very interesting for environmental applications such as the purification of wastewaters containing both organic pollutants and heavy metals.

In the research here reported, we report for the first time the preparation procedure and full physicochemical characterization of Poly-βCD/PVA sub-micrometric fibers with a dual complexation capacity, i.e., both with organic molecules and with metal ions. PVA was used as a modifier of Poly-βCD fibers to improve the mechanical properties and homogeneity of the fibers, and to reduce their diameters. Different fibrous mats were obtained by electrospinning aqueous solutions containing the hyper-branched water-soluble Poly-βCD and PVA. Long and uniform fibers with diameters in the submicron range were produced and subsequently cross-linked by a mild thermal treatment to obtain water-insoluble fibers. The cross-linking behavior with regard to the Poly-βCD/PVA ratio, the curing temperature, and duration were studied in order to optimize the post-spinning thermal treatment. In addition, the preliminary adsorption experiments of Cu^2+^ and Cd^2+^ from aqueous solutions were carried out to demonstrate the potential of the Poly-βCD/PVA fibers in wastewater treatments.

## 2. Experimental 

### 2.1. Materials

β-cyclodextrin (βCD) was kindly provided by Roquette Frères (Lestrem, France). Poly(vinyl alcohol) (PVA, Mn = 89–98.000 Da; hydrolyzed > 99%), pyromellitic dianhydride (PMDA), dimethyl sulfoxide (DMSO), ethyl ether, triethylamine, sodium hydroxide, and oxalic acid were purchased from Sigma-Aldrich. Copper(II) sulfate standard solution (Sigma-Aldrich) and cadmium standard for ICP TraceCERT^®^, 1000 mg/L Cd in nitric acid (Merck) were used for the adsorption experiments and instrumental calibration of inductively coupled plasma-optical emission spectroscopy (ICP-OES).

### 2.2. Synthesis of the Poly-βCD

First, 0.977 g of anhydrous βCD were dissolved in 6 mL of DMSO adding 1 mL of triethylamine as catalyst. After that, 2.254 g of PMDA were added under continuous stirring for 3 h at room temperature (RT). The viscosity of the solution slowly increases, but after a few hours it becomes constant. Then, the solution was precipitated in ethyl ether, solubilized in water, and then lyophilized as previously reported [33].

### 2.3. Preparation of Poly-βCD/PVA Blends

Different Poly-βCD/PVA blends (50/50 wt %, 70/30 wt %, 80/20 wt %) were prepared by dissolving the proper amounts of Poly-βCD and PVA in deionized water.

In a typical procedure, Poly-βCD and PVA are added to 3 mL of deionized water and stirred for 10 min at room temperature (RT) in closed vials. The solution is heated at 85 °C for 50 min. Then, it is left to cool to RT under continuous stirring.

The composition of the blends is reported in Table 1.

### 2.4. Preparation of Poly-βCD/PVA Fibers

A self-configured laboratory-scale electrospinning device was used to process the polymer solutions. The electric field applied between the nozzle and the collector was generated by a Glassman (Glassman High Voltage) high-voltage power supply, while a plastic syringe coupled with a volumetric pump (kd Scientific) granted a constant feed of polymer solution to the nozzle.

Solutions of the blends described in Table 1 were electrospun using a feed rate of 0.5 mL/h, a working distance of 15 cm, and an applied electrical potential of 15 kV.

Fibers were cured in a Lenton 1200 tubular furnace under nitrogen flow (60 mL/min) and by applying the following treatment: ramp of 10 °C/min from RT to 160 °C, 10 min at 160 °C.

### 2.5. Characterization

A Rheometer TA Instruments Discovery HR equipped with 20 mm plate-plate geometry was used to investigate the viscosity of the polymeric solution at 100 L/s of shear rate for 120 s at room temperature.

Fourier-transform infrared spectroscopy in the attenuated total reflectance mode (ATR-FTIR) (Perkin-Elmer Spectrum 100) was used for collecting the spectra of the fibers before and after curing. A total of 16 scans were recorded in the range from 4000 to 650 cm^−1^ at 4 cm^−1^ resolution.

Scanning electron microscopy (SEM) (Zeiss EVO XVP-LaB6) was applied to investigate the sample morphology. Samples were mounted on metallic stubs with a double-sided conductive tape and were ion-coated with gold by a sputter coater (Baltec SCD 050) for 60 s under vacuum at a current intensity of 40 mA. The diameter distribution was calculated manually using ImageJ software.

Thermal behavior and stability of the fibers were studied by thermogravimetric analysis (TGA) (TGA 2950 balance TA Inc.). First, 15 mg of sample were placed in an alumina plate and heated to 800 °C by a ramping temperature of 10 °C/min under nitrogen flow.

A Q200 (TA Instruments) differential scanning calorimeter (DSC) was used to study the thermal behavior of fibers. The DSC experiments were performed on fiber mats of about 5 mg in an aluminum crucible. The as-spun mats were studied in a heat-cool-heat cycle in the range from −50 to 195 °C with a ramp rate of 10 °C/min, under nitrogen flow. The curing process of the fibers was also studied adding an isothermal step at 160 °C, followed by cooling to 30 °C and then a second heating ramp to assess the completeness of the curing process.

The solubility tests of the as-spun fibers and cured fibers were carried out by dispersing 5 mg of each sample in 5 mL of bidistilled water under continuous stirring at RT. Then, the solutions were centrifuged and filtered using 0.45 μm PTFE filters and analyzed by using a double-beam Lambda 25 spectrophotometer (Perkin-Elmer). The analysis is performed in a quartz cuvette (optical path: 1 cm).

Contact angles were determined using the sessile drop method by the DSA100 drop shape analyzer (Krüss GmbH, Hamburg, Germany). Droplets of 6 μL of deionized water were deposited on Poly-βCD/PVA films using an automatic dispenser. The drop shape was monitored with a digital camera for 20 s, and contact angle values were calculated by DSA3 software. Measurements were performed on five positions on each sample.

The carboxyl group content per gram of polymer was estimated by titration. Specifically, 100 mg of Poly-βCD, before and after curing, was added to 30 mL of deionized water, under mild stirring and titrated in triplicate against a 0.1 N sodium hydroxide solution (standardized using a 0.1 N oxalic acid solution). All titrations were performed in an ice bath to reduce the hydrolysis of ester bonds. pH was monitored using a Hanna Instruments HI8521 pH-meter.

The adsorption of Cu^2+^ and Cd^2+^ was quantified by inductively coupled plasma-optical emission spectroscopy (ICP-OES) with a Perkin Elmer Optima 7000 DV apparatus, which was provided with an Echelle monochromator, a cyclonic spray chamber, and a Teflon Mira Mist nebulizer. The instrumental conditions were: plasma power 1.3 kW; sample aspiration rate 15 rpm (approximately 2 mL/min); argon nebulizer flow 0.6 L/min; argon auxiliary flow 0.2 L/min; and argon plasma flow 15 L/min.

The adsorption experiments were performed starting from 25 mL of 10 mg/L Cu^2+^ and Cd^2+^ distilled water solution. The pH of the solutions resulted in 4.2 in the case of Cu^2+^, while it was 2.4 in the case of Cd^2+^. The adsorption experiments were carried out at room temperature by dipping 5 mg of fibrous mat at an increasing adsorption time of 30 min, 1 h, 5 h, and 24 h. ICP-OES analyses were performed after each adsorption time on solutions previously filtered by a 0.45 µm PTFE filter and successively added by nitric acid (65 wt % – Merck Millipore). Each concentration value was averaged on the basis of three measurements. ICP-OES data are expressed in mg/g (i.e., mg of metal ion per gram of adsorbent).

## 3. Results and Discussion

**Figure 1:**
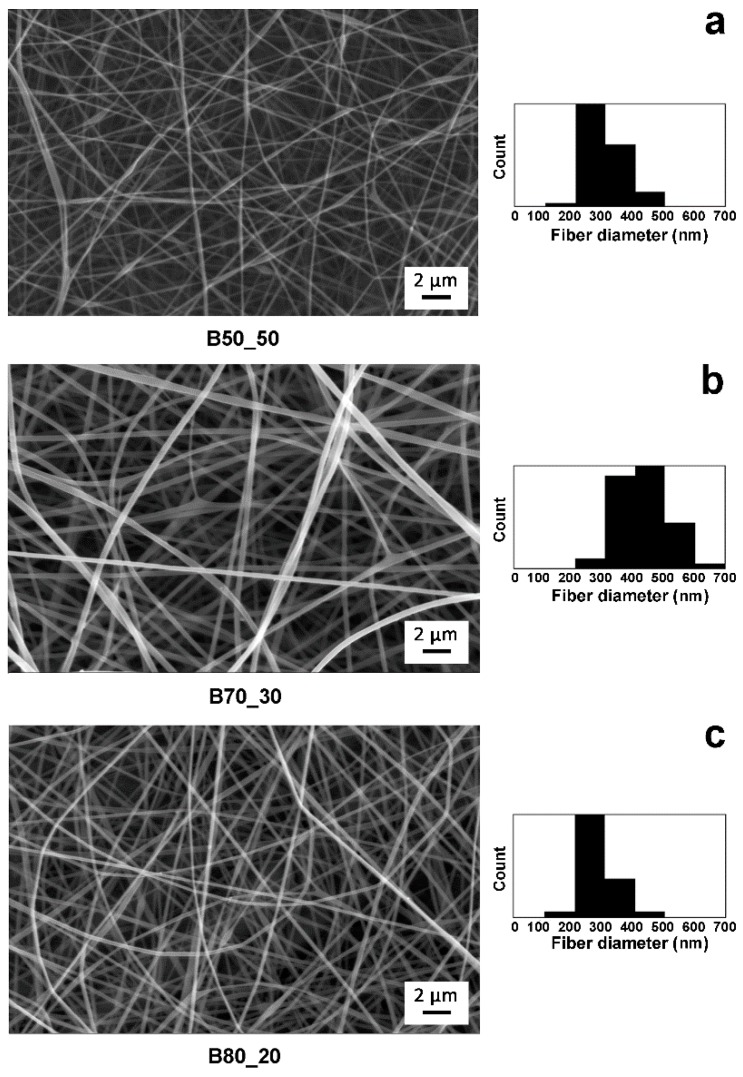
SEM micrographs of as-spun mats and histograms showing the fiber diameter distribution: B50_50 (**a**), B70_30 (**b**), and B80_20 (**c**).

Different solutions were prepared by dissolving Poly-βCD and PVA in deionized water as described in the Experimental section. All the Poly-βCD/PVA blends were highly water soluble, giving spinnable water solutions whose composition and viscosity are reported in Table 1. However, SEM images of the electrospun fibers showed different morphologies depending on the composition of the solutions. The as-spun fibers from the B50_50 solution (Figure 1a) have an average diameter of approximately 275 ± 60 nm and they are not very homogeneous in shape as there are some beads along the fibers. Beads were eliminated or very reduced by increasing the viscosity of the solutions, which leads to a reduction in surface tension. Indeed, the as-spun mats from B70_30 and B80_20 solutions reveal a good uniformity with continuous fibers without any beads. On the other hand, the relationship between viscosity and fiber size appears more unpredictable: as expected, the B70_30 solutions, being more viscous, gave fibers with an average diameter of 429 ± 80 nm, which was therefore greater than the B50_50 solutions. However, further increasing the amount of Poly-βCD and the overall viscosity of the solutions, the fiber average diameter drops to 292 ± 60 nm (sample B80_20, Figure 1c). This may be because the presence of more Poly-βCD in the solution increases the charge density and allows more stretching of the filament during the jet whipping.

**Figure 2:**
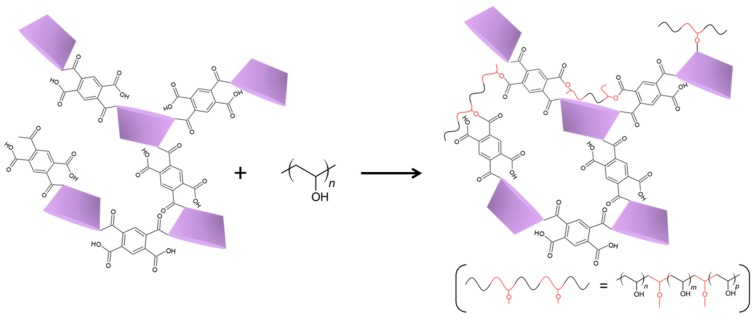
Scheme of the cross-linking reactions between Poly-βCD and PVA during the curing treatment.

**Figure 3:**
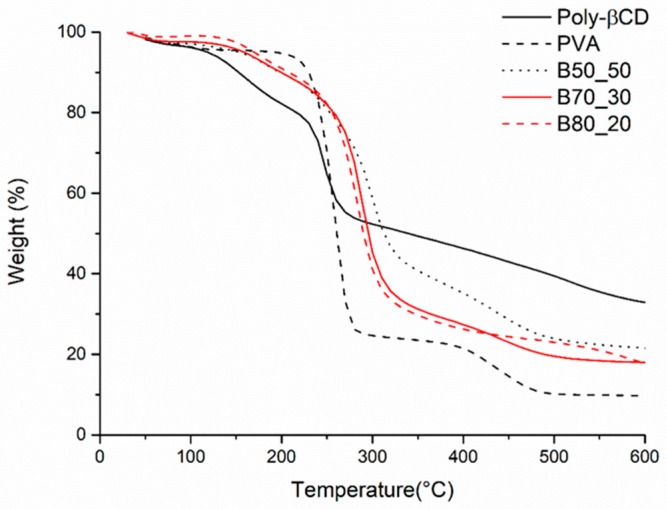
TGA curves of Poly-βCD (red solid line), PVA (red dashed line), B50_50 (black solid line), B70_30 (black dashed line), and B80_20 (black dotted line).

In order to be applied for separation/purification processes in aqueous environment, the as-spun fibers need to become insoluble. Both Poly-βCD and PVA can be thermally cross-linked by the condensation of hydroxy groups with carboxylic acids (Figure 2). In this way, the two polymers can be joined in a cross-linked structure, which provides water insolubility and also results in an improvement of the mechanical properties of the fibers. In order to find the optimal thermal treatment for curing Poly-βCD/PVA mats, a thermoanalytical study using FTIR as well was performed. Figure 3 reports the TGA curves of the three types of Poly-βCD/PVA microfibers and those of the two individual components, in powder form, for comparison. These curves show a small mass loss within the first 100 °C, which is attributable to the volatilization of adsorbed water, and then three main mass loss steps: the first starts at approximately 100 °C and ends at 200 °C, the second occurs between 200 and 310 °C, and the last mass loss is between 310 and 480 °C. The first mass loss occurs in a temperature range where PVA is stable, and it can be attributed to the residual reactivity of free carboxylate groups in the hyper-branched Poly-βCD [32]. In this step, the Poly-βCD sample loses about 25% of its initial weight, while the Poly-βCD/PVA fibers lose less than 10%, including also the loss of adsorbed water. The second main degradation step is due to the fragmentation of both PVA and Poly-βCD chains, which results in the formation of volatile fragments, and to the simultaneous cross-linking of the material by the condensation of hydroxy and carboxylate functionalities, which results in the formation of a carbonaceous residue.

**Figure 4:**
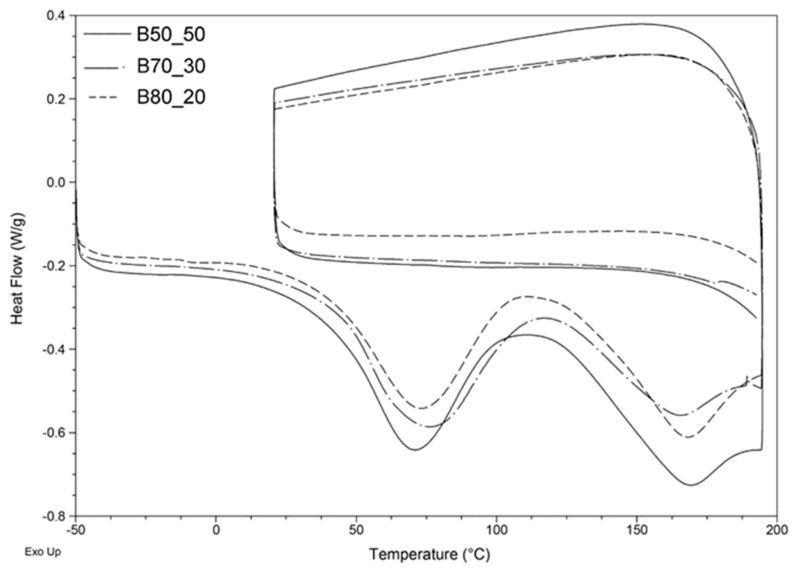
Differential scanning calorimeter (DSC) of B50_50 (solid line), B70_30 (dashed line) and B80_20 (dotted line).

To better understand the curing reaction, DSC analyses were carried out and the results obtained for the three polymer blends are reported in Figure 4. Firstly, samples were heated to 200 °C; then, they were cooled to 30 °C and heated again. During the first ramp, an exothermic process takes place between 70 and 170 °C which can be associated with a cross-linking process [34]. At the end of the first heating ramp, the cross-linking reaction can be considered complete, since no exothermic phenomena are observed in the second heating ramp. Since the curing reaction occurs for all the samples between 70 and 180 °C, it was decided to use a temperature of 160 °C as the curing temperature. Then, DSC analyses including an isothermal step at 160 °C were performed to identify the correct duration of the isothermal treatment, and it was found that 10 min was enough to guarantee the complete cross-linking of the samples (i.e., no residual cross-linking was detected on performing a second heating ramp after the isothermal treatment).

**Figure 5:**
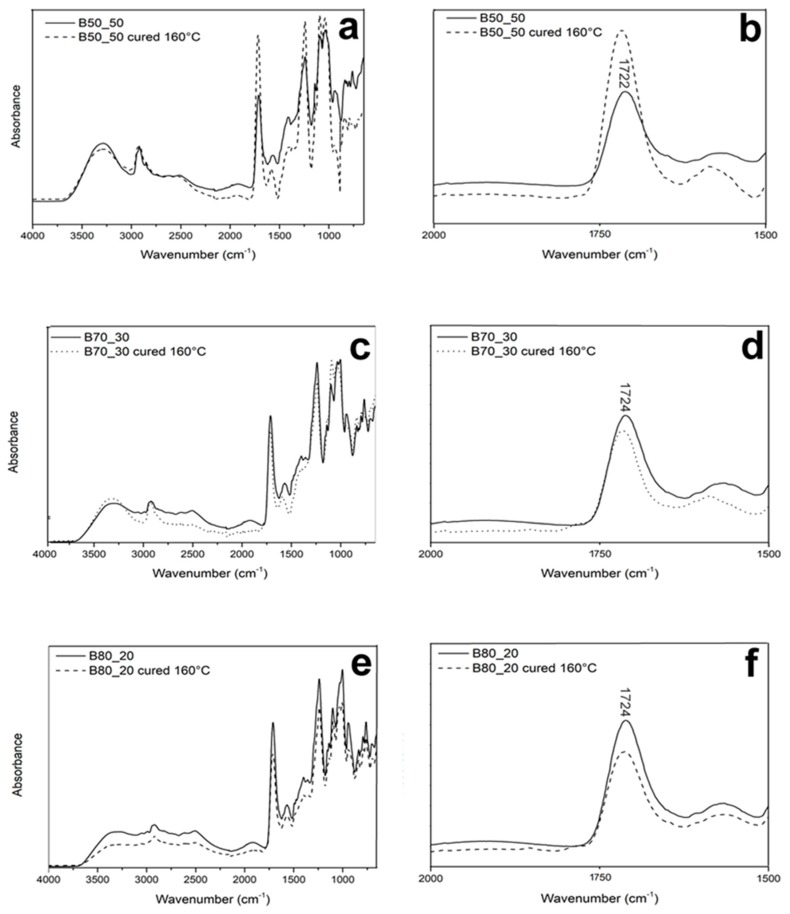
Full attenuated total reflectance mode (ATR-FTIR) spectra of as-spun and cured fibers: B50_50 (**a**), B70_30 (**c**), and B80_20 (**e**). Magnification of the C=O stretching: B50_50 (**b**), B70_30 (**d**), and B80_20 (**f**).

ATR analysis on the uncured and cured fiber mats were performed in order to identify differences in the profiles of the FTIR spectra due to the possible degradation of the material. Figure 5 shows the spectra of the uncured fibers and those cured at 160 °C. All the spectra show similar peaks. Importantly, no additional bands at 1850 cm^−1^ and 1780 cm^−1^ can be seen in the cured fibers. These bands are ascribed to the C=O stretching of pyromellitic moieties formed by the scission of the ester bridge inside the Poly-βCD [36,37]. Their absence confirms that the thermal treatment does not damage the structure of the polymer in the fibers.

**Figure 6:**
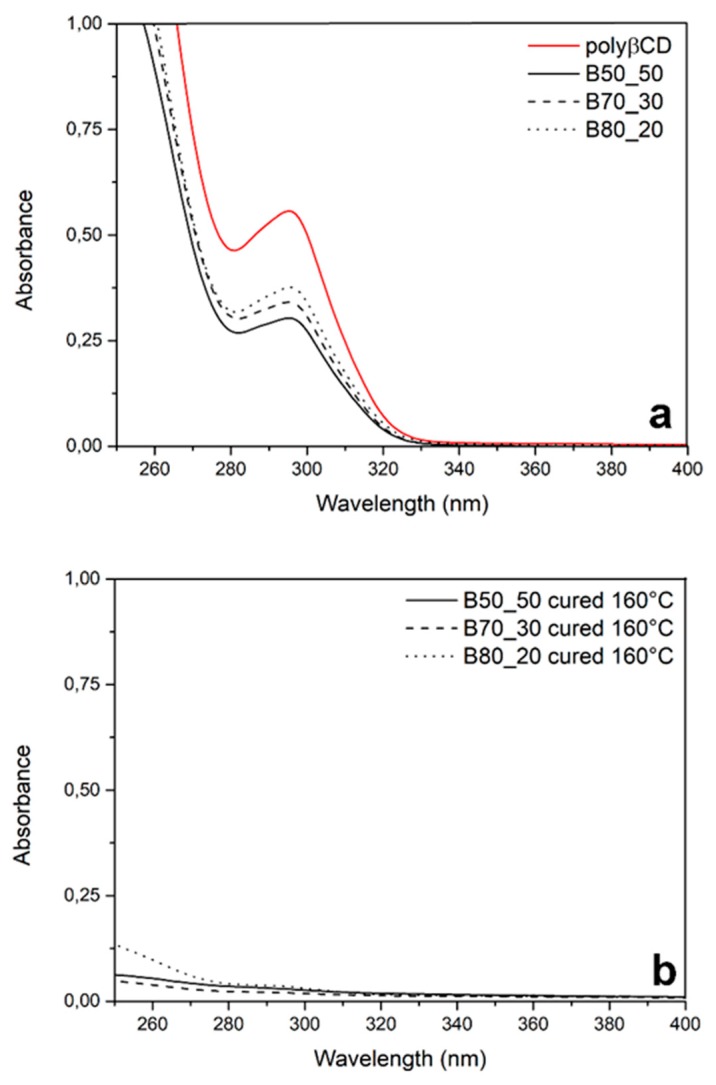
UV-vis spectra of water solutions of as-spun fibers (**a**) and of fibers cured at 160 °C (**b**).

Further details on the effectiveness of the curing treatment were obtained by UV-vis spectroscopy. The as-spun mats are perfectly water soluble, and their solutions generate an absorption band at 296 cm^−1^ in UV-vis spectra (Figure 6a). To verify the presence or not of residual fractions of soluble material in the cured fibers, fiber mats cured at 160 °C for 10 min were put in 5 mL of bidistilled water and analyzed after 24 h of continuous shaking. The resulting spectra are almost flat, confirming that the thermal treatment at 160 °C is suitable for completing the curing and getting a fully insoluble sample (Figure 6b).

**Figure 7:**
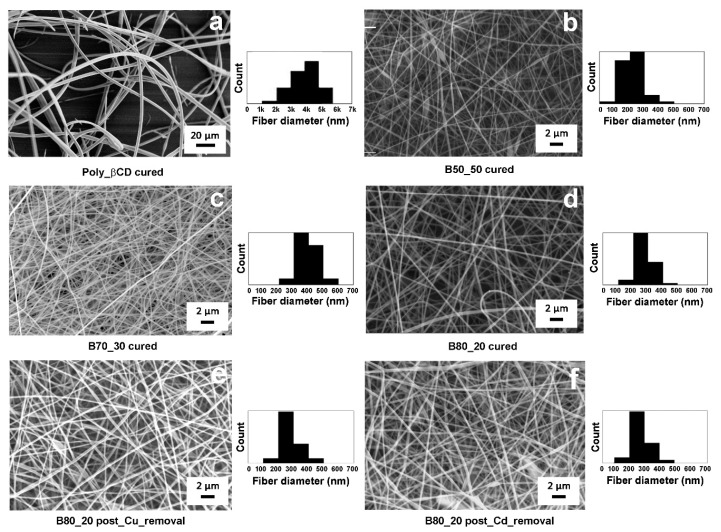
SEM micrographs of cured fibers and histograms showing the fiber diameter distribution: Poly-βCD (**a**), B50_50 (**b**), B70_30 (**c**), B80_20 (**d**), B80_20 after adsorption and desorption of copper (**e**) and cadmium (**f**).

After a curing treatment of 10 min at 160 °C, fibers were examined by SEM. Figure 7b–d show that the morphology and the dimension of the Poly-βCD/PVA fibers is not significantly affected by the post-spinning curing process. Differently from electrospun fibers of pure Poly-βCD (Figure 7a), which were previously reported by our group [34], in Poly-βCD/PVA samples, no broken fibers are observed, pointing to a more tenacious and less brittle material. This is because the presence of PVA, which at 160 °C cross-links less than the Poly-βCD, reduces the overall cross-linking density, ensuring greater mobility of the chain segments at the molecular level and therefore greater flexibility than Poly-βCD alone. This is confirmed by the DSC analyses carried out on the fibers after the curing treatment at 160 °C: While in the Poly-βCD fibers no glass transition signal is observed due to the high cross-linking density, in those containing PVA, a signal is observed at about 80 °C, relative to the glass transition of PVA, which decreases in intensity, increasing the amount of Poly-βCD. In addition, the Poly-βCD/PVA fibers are more homogenous and thinner, thus exposing a larger surface area to the solutions with which they come into contact. This is an important aspect for adsorbent materials.

In order to investigate the wettability of the fibrous mats and identify any variations in the surface properties of the fibers after curing, sessile drop contact angle measurements were carried out. As it was not possible to measure directly the contact angles of the fibrous mats because the drops of water deposited on the samples instantly penetrate through the mats, measurements were carried out on thin films prepared from the three Poly-βCD/PVA blends. The contact angles were found to be the same for all samples, within an experimental error range. In particular, the contact angles were between 57° and 52°, both for the as-spun and the cured samples, showing that the fibers of all three compositions are hydrophilic and maintain their hydrophilicity after the mild thermal cross-linking treatment. The thermal treatment does not affect significantly the content of carboxyl groups of Poly-βCD, either. As revealed by titration experiments, the initial number of carboxyl groups per gram of polymer, which is a crucial factor to the complexation capacity of the polymer, decreased by only 13% after curing (from 3.0 × 10^−3^ ± 1 × 10^−4^ mol/g to 2.6 × 10^−3^ ± 2 × 10^−4^ mol/g).

Fiber efficacy in removing heavy metals was evaluated by immersing fibrous mats in Cu^2+^ and Cd^2+^ solutions and quantifying the ions remaining in solution by ICP-OES at increasing immersion times (30 min, 1, 6 and 24 h). Moreover, in order to assess the reversibility of the adsorption process, the B80-20 samples kept in contact with Cu^2+^ and Cd^2+^ for 24 h were later analyzed after being rinsed with a 0.1 M HCl solution for 2 h. Importantly, the morphology of the fibers did not change: no swelling or fusion of the fibers was observed and even after metal ion desorption tests the fibers were still uniform and dimensionally stable (Figure 7e–f).

**Figure 8:**
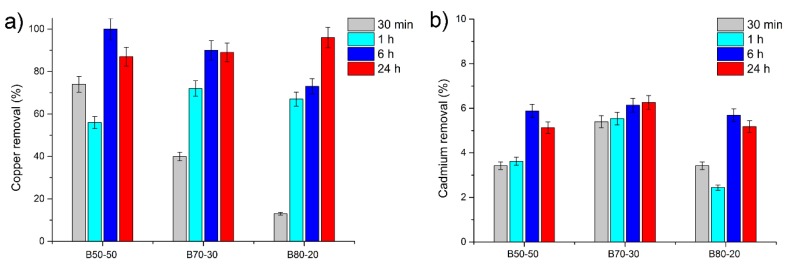
Copper (**a**) and cadmium (**b**) removal from aqueous solutions containing 10 mg/L of Cu and Cd respectively after different contact times (30 min, 1 h, 6 h, and 24 h).

ICP-OES data were normalized according to each sample weight and are reported in Figure 8. With regard to copper, the B80_20 fibers gradually adsorb Cu^2+^ with the increasing contact time. On the contrary, the B70_30 samples show a maximum retention of copper after only 6 h of contact (Figure 8a). Indeed, the B70_30 sample shows a weak decrease in the adsorption trend (of the order of 0.14 mg/L) between 6 and 24 h of contact. This negligible reduction could be associated to a slight inhomogeneity in the sample used or to an unbalancing in the equilibrium of the system. The B50_50 fibers show a good performance in terms of copper adsorption, although the adsorption trend as a function of contact time is irregular and difficult to rationalize. ICP-OES analyses performed on B80_20 fibers after 24 h of contact with the Cu^2+^ solution and later immersed in a strong acidic solution show that the adsorbed copper is almost fully released and active sites are regenerated. Overall, after 24 h of adsorption, considerably high values of copper adsorption capacity were obtained for all the three Poly-βCD/PVA fibers, ranging from 43.73 mg/g for B50_50 fibers to 48.15 mg/g for B80_20 fibers. These performances are very interesting, superior, or comparable with those reported in other researches about new sorbents, especially considering that the values here obtained refer to an initial metal ion concentration of 10 ppm. Such a low concentration was chosen to test the adsorption capacity of the fibers in critical conditions for metal ion complexation. In fact, the Cd and Cu concentrations in wastewater samples are usually in the order of mg/L. In addition, according to literature data, the optimal concentration that allows getting the best adsorption of metal ions per gram of adsorbent is usually higher than 10 mg/L [27,38]. Similarly, the adsorption experiments were carried out at acid pH to evaluate the efficiency of wastewater treatment in extreme conditions.

Differently from Cu^2+^, the adsorption efficiency of the sample set kept in contact with Cd^2+^ does not exceed a value of 1 mg/L in any case (Figure 8b). Depending on the composition of the fibers, there are small differences in their capacity to adsorb Cd^2+^, but the amount of Cd removed from the aqueous solutions, even after 24 h of contact, is always less than 10%.

The different retention of the two metal ions partially confirms what emerged from a previous research by Berto et al. [31] in which the complexation mechanisms involved in the adsorption of heavy metals on a cross-linked polymer based on β-CD and pyromellitic anhydride were investigated. This study suggests that the complexation of Cu^2+^ by the CD-based cross-linked polymer does not depend only on the presence of carboxylate groups, but also involves the alcoholate groups on the cyclodextrin moieties. On the contrary, in the case of Cd^2+^, the interaction with the polymer essentially occurs by complexation with carboxylates, without the involvement of other sites. Consequently, the retention of Cd^2+^ is lower than that of Cu^2+^. In addition, the role of PVA in the complexation of the metal ions has also to be taken into account. As reported by Hosny et al., the Cu(PVA) complex starts to form at pH ≈ 5 and reaches its maximum percentage at pH ≈ 8, while the Cu(PVA)_2_ complex starts to form at pH ≈ 7.8 [39]. Furthermore, this study confirms that the stability of Metal–PVA complexes of metal ions of the same charge is inversely proportional to metal ion radii, which is reflected in fact in the lower capacity of the poly (β-CD)/PVA fibers to remove Cd^2+^ compared to Cu^2+^.

Thus, the greater adsorption capacity of the Poly-βCD/PVA fibers for Cu^2+^ rather than for Cd^2+^ is coherent with what has been reported by other authors [31,40], even if more in-depth studies are necessary to clarify the performances of Poly-βCD/PVA fibers and the mechanisms involved in the adsorption of heavy metals.

## 4. Conclusions

Blends of the hyper-branched Poly-βCD and PVA were successfully electrospun to give sub-micrometric Poly-βCD/PVA sub-micrometric fibers. These were made insoluble by a post-spinning thermal treatment which leads to the co-cross-linking of the two polymers by a reaction between hydroxyl and carboxyl functionalities and to the formation of a single polymer network where the presence of PVA segments makes the poly-βCD-based fibers less brittle. PVA also facilitates the processing of Poly-βCD by electrospinning, allowing obtaining fibers with variable composition, with a more homogeneous morphology, and with a greater surface area than those that can be obtained from Poly-βCD-only solutions. The possibility of using these fibers for wastewater treatments was explored by testing the adsorption of Cu^2+^ and Cd^2+^. ICP-OES data demonstrate that Poly-βCD/PVA fibers can complex both metal ions and are especially efficient adsorbents of Cu^2+^. All the different types of Poly-βCD/PVA fibers can remove more than 50% of Cu^2+^ after 1 h of contact, reaching or exceeding 85% after 6 h.

These results lay the foundations for the future production of poly-βCD/PVA fibers and membranes that are capable of forming inclusion complexes with organic molecules and at the same time removing heavy metals.

## Figures and Tables

**Table 1:** Properties of poly(β-cyclodextrin) (Poly-βCD)/poly(vinyl alcohol) (PVA) solutions and average diameters of the corresponding fibers.

Sample Name	Poly-Βcd (mg)	PVA (mg)	% Poly-βCD (w/v)	% PVA (w/v)	% Polymer (w/v)	Viscosity (Pa·s)	Fiber Diameter (nm)
*B50_50*	225	225	7.5	7.5	15.0	0.164	275 ± 60
*B70_30*	525	225	17.5	7.5	25.0	0.387	429 ± 80
*B80_20*	900	225	30.0	7.5	37.5	0.562	292 ± 60
